# HiJAKing Immunotherapy-Resistant Melanoma for a Cure

**DOI:** 10.1093/oncolo/oyac270

**Published:** 2023-01-14

**Authors:** Lewis Zhichang Shi, Hongxing Shen, Oluwagbemiga A Ojo, James A Bonner

**Affiliations:** Department of Radiation Oncology, Heersink School of Medicine at the University of Alabama at Birmingham (UAB-SOM), Birmingham, AL, USA; O’Neal Comprehensive Cancer Center, University of Alabama School of Medicine, Birmingham, AL, USA; Department of Microbiology, University of Alabama School of Medicine, Birmingham, AL, USA; Department of Pharmacology and Toxicology, University of Alabama School of Medicine, Birmingham, AL, USA; Programs in Immunology, University of Alabama School of Medicine, Birmingham, AL, USA; Department of Radiation Oncology, Heersink School of Medicine at the University of Alabama at Birmingham (UAB-SOM), Birmingham, AL, USA; Department of Radiation Oncology, Heersink School of Medicine at the University of Alabama at Birmingham (UAB-SOM), Birmingham, AL, USA; Department of Radiation Oncology, Heersink School of Medicine at the University of Alabama at Birmingham (UAB-SOM), Birmingham, AL, USA; O’Neal Comprehensive Cancer Center, University of Alabama School of Medicine, Birmingham, AL, USA

**Keywords:** JAK inhibition (JAKi), immune checkpoint blockers, melanoma

## Abstract

Immune checkpoint blockers (ICBs) have brought great promise to patients with advanced melanoma, a tumor type that was claimed largely incurable not long ago. However, therapeutic resistance to ICBs has limited their utility in the clinic. Here, we provide a commentary on recent research endeavors concerning ICB resistance in melanoma patients.

## Teaser

This commentary highlights recent research endeavors concerning immune checkpoint blocker resistance in patients with melanoma.

Skin cancer is the most common cancer type, outnumbering all other types of cancer combined. Although only ~1% of all skin cancers are melanoma, it is the deadliest form and accounts for most skin cancer-related deaths. Prior to 2011, no therapeutics had induced a survival advantage in patients with advanced melanoma. The dire situation was changed in 2011 when the U.S. Food and Drug Administration (FDA) approved ipilimumab, an anti-human CTLA-4 monoclonal antibody that successfully improved the survival of melanoma patients in phase III clinical trials,^1^ opening a new era of immunotherapy on ICBs. By releasing immune “brakes” (ie, CTLA-4, PD-1/L1) that tumor cells co-opt to suppress T cells, ICBs rejuvenate tumor-infiltrating T cells (TILs),^2-4^ leading to tumor rejection. To date, more than 70 approvals have been granted to ICBs to treat various types of cancer,^5^ some of which are for first-line use, establishing ICBs as a major pillar for cancer care. Despite these transformative clinical benefits, accumulating data indicate that therapeutic resistance to ICBs is common and only a small subset of patients are responsive to ICBs. Therefore, deciphering the underlying mechanisms of resistance to ICBs and then overcoming them are of pressing importance to improve the overall efficacy of ICBs in melanoma patients.

Pioneering work from the Schreiber group showed that tumors lacking functional IFN-γ signaling can escape immunosurveillance and fail to induce memory response,^6,7^ highlighting a pivotal role of tumor-intrinsic IFN-γ signaling in orchestrating endogenous anti-tumor response. To explore its role in the setting of ICBs, the Ribas group from UCLA analyzed melanoma samples from patients that developed therapeutic resistance to anti-PD-1 and found that loss of JAK1 and JAK2 (2 essential downstream kinases in the IFN-γ signaling pathway) in melanoma cells confers resistance to anti-PD-1.^8^ Independently, using a cohort of patients with advanced melanoma treated with anti-CTLA-4, the Sharma group from MD Anderson Cancer Center reported that ~75% of patients were not responsive to anti-CTLA-4 and their tumors harbored copy losses of IFN-γ signaling genes.^9^ They further confirmed that knockdown of the IFN-γ signaling (IFNγR1^KD^) in syngeneic B16-BL6 melanoma cells rendered them less sensitive to anti-CTLA-4 but did not disclose detailed information on the specific mutations of the IFN-γ signaling genes. To this end, a later study using a 12-member core gene set from the IFN-γ signaling pathway (ie, *IFNGR1*, *IFNGR2*, *JAK1*, *JAK2*, *JAK3*, *TYK2*, *STAT1*, *STAT2*, *STAT3*, *STAT5A*, *STAT5B*, and *STAT6*) reported that the cumulative frequency of their damaging mutations (eg, gene deletion and deleterious mutations) is 20.9% among 287 cases of skin cutaneous melanoma (SKCM) (http://www.cbioportal.org/data_sets.jsp).^10^ In a separate study, another group established 46 melanoma cell lines using metastases collected from different melanoma patients and observed that 6 of them had mutations in *JAK1* (4 lines), *JAK2* (1 line), or *STAT1* (1 line).^11^ Among these 6 lines, 3 of them had homozygous mutations, coupled with loss of or greatly impaired IFN-γ signaling. Using a gene set of 6 members in the IFN-γ signaling pathway (*IFNGR1*, *IFNGR2*, *JAK1*, *JAK2*, *STAT1*, and *IRF1*), this group also mined the aforementioned 287 melanoma tissue samples and identified their mutations in 12.6% of them (36 cases), among which a decent fraction (44%, 16 of 36) had homozygous deletions in different genes, indicating a dysfunctional IFN-γ signaling. In spite of the differences between these 2 studies, they nevertheless congruently demonstrate that damaging mutations in IFN-γ signaling pathway genes are present in a considerable number of melanomas, predisposing them to develop resistance to IFN-γ and ICBs. Of note, given the essential role of TILs in dictating ICB efficacy,^2-4^ the Sharma study did not reveal overt changes of TILs in IFNγR1^KD^ melanoma,^9^ likely because IFNγR1^KD^ cells still possessed some degree of the IFN-γ signaling. Therefore, it remains to be established whether and how melanoma IFN-γ signaling modulates TILs and regulates ICB response. Consequently, strategies of how to overcome the ICB resistance associated with the loss of IFN-γ signaling in melanoma cells have been largely elusive.

To address these issues, we generated a melanoma model with knockout of the IFN-γ signaling by deleting IFNγR1 from B16-BL6 cells using the CRISPR-Cas9 technology (IFNγR1^KO^).^12^ We first confirmed that IFNγR1^KO^ cells were defective of IFN-γ signaling, reflected by their lack of IFN-γ-induced upregulation of IRF-1 (a direct transcriptional target of IFN-γ), PD-L1 expression, and cell killing. Moreover, IFNγR1^KO^ melanoma was completely resistant to anti-CTLA-4, consistent with the reduced sensitivity of IFNγR1^KD^ melanoma to anti-CTLA-4 treatment.^9^ Using this “clean” model, our first interesting finding was that unlike IFNγR1^KD^ melanomas that had largely normal TILs,^9^ IFNγR1^KO^ melanomas had much reduced abundance of CD8^+^ TILs at the baseline and did not show increased infiltration and functional rejuvenation of TILs upon anti-CTLA-4, establishing an active role of melanoma IFN-γ in shaping TILs. In direct correlation with this preclinical finding, bioinformatic analyses of human SKCMs with impaired IFN-γ signaling (IFNGR1^Low^ SKCMs) also showed reduced expression of T-cell signature genes. This negative regulation of TILs by melanoma loss of the IFN-γ signaling, together with the reduced sensitivity of IFNγR1^KO^ melanoma cells to IFN-γ-mediated tumor killing, poses a dual resistance to ICBs.

**Figure 1.**
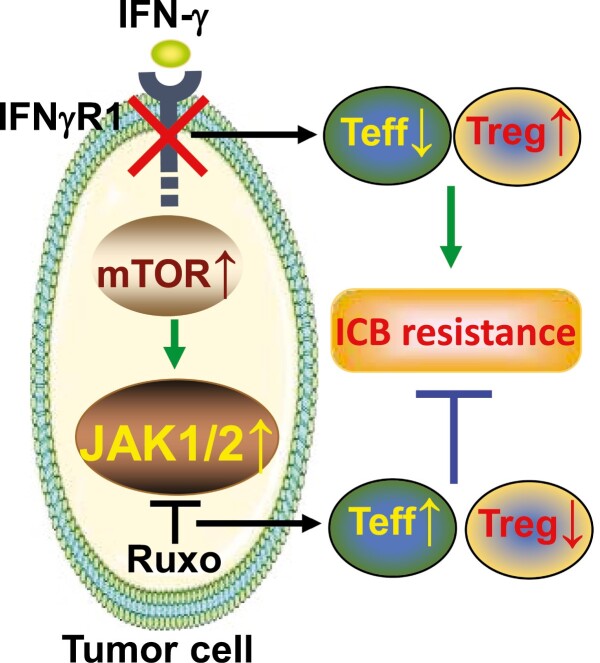
The mTOR-JAK1/2 is a therapeutic target to overcome ICB resistance in melanoma defective of IFN-γ signaling. Abbreviations: IFN γ R1^KO^, knockout of the essential IFN- γ receptor 1; mTOR, the mechanistic target of rapamycin, a master regulator of cellular metabolism; JAK1/2, Janus kinases 1&2, essential kinases in the IFN-γ signaling; Ruxo, Ruxolitinib, an FDA-approved JAK1/2 inhibitor; T_eff_, effector T cells (eg, TNF-producing T cells); T_reg_, FoxP3^+^ regulatory T cells; ICB: immune checkpoint blocker.

Next, we wanted to uncover therapeutic targets that can be harnessed to overcome ICB resistance in IFNγR1^KO^ melanoma. To do this, we took a multi-omics approach encompassing RNA-seq, kinomics, and phosphoproteomics. To our surprise, these analyses revealed a network of constitutively active protein tyrosine kinases (PTKs) centered on JAK1/2 in IFNγR1^KO^ cells, mediated by the heightened mTOR signaling pathway. We further confirmed this unexpected finding by directly analyzing phosphorylation of these kinases with Western blot. To establish the clinical relevance, we conducted analyses of IFNGR1^Low^ SKCMs and ICB-resistant patient melanomas, which also showed expected changes of target genes regulated by mTOR and JAK1/2, indicating a similar activation of the mTOR-JAK axis in human melanomas with impaired IFN-γ signaling and ICB resistance. To explore if activated JAK1/2 could serve as attractive therapeutic targets for ICB resistance, we treated melanoma-bearing mice with Ruxolitinib (Ruxo), an FDA-approved JAK1/2 inhibitor for myeloproliferative neoplasms (MPN) and other pathologies. Commensurate with activated JAK1/2 in ICB-resistant IFNγR1^KO^ melanoma, Ruxo selectively suppressed IFNγR1^KO^ but not scrambled control melanomas, highlighting Ruxo as a “targeted” therapy for ICB resistance. Mechanistically, Ruxo effects were not due to its preferential killing of IFNγR1^KO^ cells but rather its prominent reprogramming of TILs. Specifically, Ruxo markedly decreased regulatory T cells (T_reg_) and concomitantly increased TNF production by CD4^+^ TILs. As such, deletion of T cells or host TNF signaling completely abolished Ruxo efficacy (Fig. 1).

In summary, we demonstrate that loss of IFN-γ signaling in melanoma hampers infiltration and functional rejuvenation of TILs, which in turn mediates ICB resistance. By inhibiting the aberrantly active JAK1/2, Ruxo reprograms the “cold” TILs and selectively suppresses IFNγR1^KO^ but not scrambled control melanoma, offering a potential “targeted” therapy for ICB resistance. Interestingly, the Ribas group recently reported that intratumoral injection of toll-like receptor 9 agonist together with anti-PD-1 engaged CD8^+^ T cells and natural killer (NK) cells to mediate the therapeutic effects in anti-PD-1-resistant JAK1/2 knockout melanoma.^13^ That study, together with our finding of an essential role of TILs in Ruxo therapy, accentuates that effective engagement of tumor-reactive T cells is the key to overcoming ICB resistance, even in melanomas lacking IFN-γ signaling, likely through other effector mechanisms involving TNF, GzmB, Perforin, CD107a, etc. To this end, we showed that TNF played an indispensable role in governing Ruxo efficacy in IFNγR1^KO^ melanoma, in line with a recent report showing an enriched TNF pathway gene signature in human IFNγR1^KO^ melanoma clones that are sensitive to T cell killing.^14^ Taken together, T cells can utilize armamentarium other than IFN-γ to drive anti-tumor immunity against melanoma lacking IFN-γ signaling and how to strategically engage them (eg, TNF) would be instrumental to overcome ICB resistance.

## Concluding Remarks

JAK inhibition with Ruxo or itacitinib (a specific JAK1 inhibitor) has been tested in patients with advanced solid tumors (NCT02646748 and NCT02646748), non–small cell lung cancer (NCT02917993 and NCT03425006), and triple-negative breast cancer (NCT02876302 and NCT03012230),^15^ but its utility in overcoming ICB resistance has not been assessed. Our results justify further testing of Ruxo in patients with advanced melanoma that are resistant to ICBs, particularly those with impaired IFN-γ signaling. While Ruxo, with its dual roles in reprogramming “cold” TILs and selective suppression of IFNγR1^KO^ melanoma, appears to be an ideal “two birds, one stone” strategy, we reason that it still needs to be combined with other therapeutics to achieve long-term cure, considering its on-target suppressive effects on MPN-associated splenomegaly that could incite potential toxicity on mature T cells.^16^ Along this line, preclinical studies from us and others have shown that Ruxo can improve therapeutic efficacy of radiotherapy,^17,18^ and when combined with oncolytic virus immunotherapy, can induce synergistic effects in different types of cancer.^19^ Based on these results, we are designing a clinical trial to test Ruxo, either as a standalone therapy or in conjunction with other therapeutic modalities, in patients with ICB-resistant melanoma. In support of this idea, there is an ongoing clinical trial combining Ruxo with the standard of care therapies (temozolomide and radiation) to treat patients with grade III gliomas and glioblastoma (NCT03514069), tumor types that are known to be resistant to ICBs. Furthermore, a preclinical study recently showed that selective blocking of JAK2 but not both JAK1 and 2 augments anti-PD-L1 therapy,^20^ which may warrant further testing in the clinic.

We are cognizant that loss of IFN-γ signaling is one of the mechanisms of resistance to ICBs, which can be derived from other intrinsic alterations in tumor cells (such as active β-catenin and PTEN loss), stroma factors (eg, infiltration of immunosuppressive *T*_reg_ and myeloid-derived suppressor cells, as well as expression of multiple inhibitory checkpoints (CTLA-4, PD-1/PD-L1, LAG-3, TIGIT, TIM-3, VISTA, etc.), and host characteristics (eg, microbiota composition). Detailed discussion of them is beyond the scope of this brief commentary, but we contemplate that the ultimate solution to overcome these seemingly distinct mechanisms of ICB resistance lies in how to effectively mobilize T-cell-mediated anti-tumor immunity, be it making tumor cells more visible to T cells through radiation and/or targeted chemotherapy, promoting T-cell infiltration through vaccination and normalization of distorted tumor vasculature, and/or enhancing T-cell function by co-inhibition of multiple inhibitory checkpoints/adoptive transfer of super-T cells (eg, CAR-T and TCR-T cells). There are excellent reviews on these topics, which are unfortunately not cited here due to the limited number of citations allowed in this brief commentary.
